# The Dead Alive

**Published:** 1868-10

**Authors:** 


### The Dead Alive.

Here is something gay, in the way of inventions. You recollect Edgar Poe's catalepsy coffin, with inside cushions for comfort, and springs for the moment of waking. The idea was very elementary and perhaps practical. But a Frenchman has beaten it all to pieces. He calls his invention a "Respiratory-Advertising Apparatus for Precipitate Inhumations." You can see the mechanism of the thing from where you are. "You can breathe while notifying the outside world that you are resurrected." What *naiveté!* By this invention the buried individual puts himself in communication with the living by means of a tube fixed over the mouth with a funnel-shaped mouth-piece, the other end projecting from the earth or stone above. "If the individual," to quote the prospectus, "finds himself uneasy in his position he has only to demand the attention of the guardians of the cemetery, which he can easily do, and his case will be attended to at once."

So that if this ingenious invention comes into general use, the people who select the cemeteries as a place of resort, must not be surprised hereafter at hearing queer sounds from time to time proceeding from the earth around them. We can imagine the surprised promenader exclaiming to a guardian: "What! you allow people to play the trombone here?" and the guardian replying: "That's no trombone. It's the old fellow of yesterday—down there—the seventh to the left—who demands a change of base!"

The inventor thinks no family ought to be without one of his tubes. The charming man! Pretty soon he will pretend that children cry for them.—*Paris Cor. N. Y. Times.*—*New York Med. Journal.*

